# Supplementary material for: The Effects of Whole-Body Vibration on Spasticity in Stroke: A Systematic Review and Meta-Analysis
Source: J Clin Med. 2025 Aug 23;14(17):5966. doi: 10.3390/jcm14175966 (PMC12428902; doi:10.3390/jcm14175966)
Supplement: Supplementary file 1 [file jcm-14-05966-s001.zip › jcm-3806909_Table S1.pdf]

Table S1. List of excluded studies and reasons for exclusion

No.	Study	Reason for exclusion
No control group		
**1**	Liepert, J., & Binder, C. (2010). Vibration-induced effects in stroke patients with spastic hemiparesis–a pilot study. Restorative neurology and neuroscience, 28(6), 729-735.	Single-group or pilot study without a control group.
**2**	Miyara, K., Matsumoto, S., Uema, T., Hirokawa, T., Noma, T., Shimodozono, M., & Kawahira, K. (2014). Feasibility of using whole body vibration as a means for controlling spasticity in post-stroke patients: a pilot study. Complementary therapies in clinical practice, 20(1), 70-73.	
**3**	Constantino, C., Galuppo, L., & Romiti, D. (2014). Efficacy of mechano-acoustic vibration on strength, pain, and function in poststroke rehabilitation: a pilot study. Topics in Stroke Rehabilitation, 21(5), 391-399.	
**4**	Yukawa, Y., Higashi, T., Minakuchi, M., Naito, E., & Murata, T. (2024). Vibration-Induced Illusory Movement Task Can Induce Functional Recovery in Patients With Subacute Stroke. Cureus, 16(8).	
**5**	Shen, X., Yu, Y., Xiao, H., Ji, L., & Wu, J. (2023). Cortical activity associated with focal muscle vibration applied directly to the affected forearm flexor muscle in post-stroke patients: an fNIRS study. Frontiers in Neuroscience, 17, 1281160.	
**Data unavailable**		
**6**	Ahn, J. Y., Kim, H., & Park, C. B. (2019). Effects of Whole-Body Vibration on Upper Extremity Function and Grip Strength in Patients with Subacute Stroke: A Randomised Single-Blind Controlled Trial. Occupational therapy international, 2019(1), 5820952.	Did not include any spasticity outcome measure such as MAS or MTS.
**7**	Annino, G., Alashram, A. R., Alghwiri, A. A., Romagnoli, C., Messina, G., Tancredi, V., ... & Mercuri, N. B. (2019). Effect of segmental muscle vibration on upper extremity functional ability poststroke: A randomized controlled trial. Medicine, 98(7), e14444.	
**8**	Kim, J. W., & Lee, J. H. (2021). Effect of whole-body vibration therapy on lower extremity function in subacute stroke patients. Journal of exercise rehabilitation, 17(3), 158.	
**9**	Abit Kocaman, A., Önal, B., Sertel, M., & Karaca, G. (2023). The effect of local vibration applied to the forearm extensor muscles on hand function and muscle activation in stroke patients: a randomized controlled study. Acta Neurologica Belgica, 123(5), 1957-1964.	
**10**	Liao, L. R., Ng, G. Y., Jones, A. Y., Huang, M. Z., & Pang, M. Y. (2016). Whole-body vibration intensities in chronic stroke: a randomized controlled trial. Medicine and science in sports and exercise, 48(7), 1227-1238.	
**11**	Park, S., & Jeong, H. (2023). Effects of Vibration Exercise on Shoulder and Knee Joints Range of Motion in Stroke Patients. Journal of International Academy of Physical Therapy Research, 14(1), 2758-2766.	
**Irrelevant comparison**		
**12**	Wang SF, Wang SL, Li CJ, Ran X. Efect of whole body vibration training combined with electromyographic biofeedback therapy on rehabilitation for stroke patients with hemiplegia. J Nurs Sci. 2021;36:16–8. https://doi.org/10.3870/j.issn.1001-4152.2021.09.016.	WBV administered in combination with another active intervention, preventing
**13**	Xiao, L., Liu, C., Li, Y., Deng, Y., Xie, B., Lin, F., & Xiao, H. (2022). Effects of whole body vibration combined with extracorporeal shock wave therapy on spasticity and balance gait parameters in hemiplegic patients	

	with stroke. Zhong nan da xue xue bao. Yi xue ban= Journal of Central South University. Medical Sciences, 47(6), 755-761.	isolation of WBV effect.
**Congress papers**		
**14**	Staderini, E. M., & Mugnaini, S. (2011, March). Personal vibrotactile stimulator for rehabilitation of the hand in stroke and Parkinson patients. In 2011 5th International Symposium on Medical Information and Communication Technology (pp. 162-166). IEEE.	Conference proceeding or abstract only; insufficient methodological and outcome details for inclusion.
**15**	Bulloch, L. (2018, July). Focal muscle vibration for post-stroke rehabilitation: A systematic review of protocols and outcomes. In Rehabilitation Engineering and Assistive Technology Society of North America (RESNA) Annual Conference. RESNA.	
**Non-English language publication**		
**16**	Eun Young Yoo, Ji-Hyuk Park, Kim Young-jo, Kim Jae-nam, Jo Sang-yun, Lee Bo-mi, & Kim Seon-ho (2014). The Effect of Vibratory Stimulation on Upper Function Recovery in Patients With Stroke. Korean Journal of Occupational Therapy, 22(1), 109-124.	Full text published in a language other than English; no English translation available.
**17**	Kim, J. H. (2020). Effects of whole body vibration exercise on lower extremity muscle activity and gait ability in stroke patients. The Journal of Korean Academy of Orthopedic Manual Physical Therapy, 26(2), 29-36.	
